# DNA Methylation of *PITX2* and *PANCR* Is Prognostic for Overall Survival in Patients with Resected Adenocarcinomas of the Biliary Tract

**DOI:** 10.1371/journal.pone.0165769

**Published:** 2016-10-31

**Authors:** Barbara Uhl, Dimo Dietrich, Vittorio Branchi, Alexander Semaan, Pauline Schaefer, Heidrun Gevensleben, Babak Rostamzadeh, Philipp Lingohr, Nico Schäfer, Jörg C. Kalff, Glen Kristiansen, Hanno Matthaei

**Affiliations:** 1 Institute of Pathology, University of Bonn, Bonn, Germany; 2 Department of Otolaryngology, Head and Neck Surgery, University Hospital Bonn, Germany; 3 Department of Surgery, University of Bonn, Bonn, Germany; 4 Department of Neuroradiology, Katharinenhospital, Klinikum Stuttgart, Stuttgart, Germany; University of South Alabama Mitchell Cancer Institute, UNITED STATES

## Abstract

Biliary tract cancers (BTC) are rare but highly aggressive malignant epithelial tumors. In order to improve the outcome in this lethal disease, novel biomarkers for diagnosis, prognosis, and therapy response prediction are urgently needed. DNA promoter methylation of *PITX2* variants (*PITX2ab*, *PITX2c)* and intragenic methylation of the *PITX2* adjacent non-coding RNA (*PANCR*) were investigated by methylations-specific qPCR assays in formalin-fixed paraffin-embedded tissue from 80 patients after resection for BTC. Results were correlated with clinicopathologic data and outcome. *PITX2* variants and *PANCR* showed significant hypermethylation in tumor vs. normal adjacent tissue (*p <* 0.001 and *p* = 0.015), respectively. In survival analysis, dichotomized DNA methylation of variant *PITX2c* and *PANCR* were significantly associated with overall survival (OS). Patients with high tumor methylation levels of *PITX2c* had a shorter OS compared to patients with low methylation (12 vs. 40 months OS; HR 2.48 [1.38–4.48], *p* = 0.002). In contrast, *PANCR* hypermethylation was associated with prolonged survival (25 vs. 19 months OS; HR 0.54 [0.30–0.94], *p* = 0.015) and qualified as an independent prognostic factor on multivariate analysis. The biomarkers investigated in this study may help to identify BTC subpopulations at risk for worse survival. Further studies are needed to evaluate if *PITX2* might be a clinically useful biomarker for an optimized and individualized treatment.

## Introduction

Biliary tract cancers (BTC) represent a heterogenous group of malignancies that include adenocarcinomas of the intra- and extrahepatic bile ducts, the ampullary region, and the gallbladder. With less than 3% of all newly diagnosed malignancies in adults in the United states, BTCs are rare neoplasms [1] that share a particularly aggressive biological behavior. Even after treatment with curative intention, the prognosis is generally poor with 5-year survival rates of <30% depending on the BTC subtype and stage of disease [2–4]. In recent years, new insights into the molecular pathogenesis of BTC have been gained by high throughput ‘omics’ technologies. Exomic sequencing, for instance, has illuminated the molecular landscapes of cholangiocarcinoma and gallbladder cancer and their potential for novel diagnostic applications and targeted therapies [5,6].

**Fig 1:**
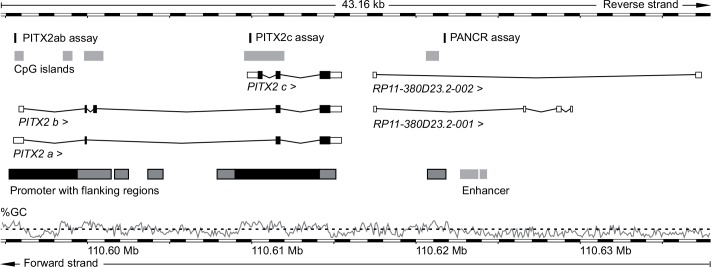
The region on chromosome 4q25 encompassing the *PITX2* gene loci and downstream the *PITX2* adjacent noncoding RNA (*PANCR*). Arrow heads indicating direction of transcription on reverse strand. Methylation assay locations are highlighted in black and CpG islands in light grey boxes. Promoter with flanking regions are displayed as black and grey and black bordered boxes, respectively. Percentage of GC nucleotides is shown with black dashed line indicating 50% GC content. Figure information is taken from Genome Reference Consortium Human Build 38 patch release 3 (GRCh38.p3), Chromosome 4: 110,594,745–110,637,909, illustrated by http://www.ensembl.org

DNA methylation is an epigenetic mechanism involved in various fundamental biological processes including cell differentiation and cell development [7,8]. Furthermore, aberrant DNA methylation has been shown to play a crucial role during carcinogenesis [9,10]. Due to the presence of cancer-specific epigenetic alterations and the considerable biostability of DNA methylation, the capacity of methylation as a biomarker has been strongly implicated [11]. The evolutionary conserved gene pituitary homeobox 2 (*PITX2)* encodes for transcription factors involved in pattern formation, genesis of several organs (e.g. heart, lungs, pituitary gland), and the determination of left-right asymmetry during embryonic development [12,13]. Mutations in *PITX2* lead to Axenfeld-Rieger syndrome, which is associated with malformations of the anterior segment of the eye [14,15]. In humans, three predominant *PITX2* isoforms transcribed from two alternative promoters sites (P1 and P2) have been identified [16,17] (Fig 1). The variants *PITX2a* and *PITX2b* are alternatively spliced transcripts originating from promoter P2, which is known to be regulated by the WNT pathway [18]. The *PITX2c* variant is transcribed from an alternative promoter P1, which is regulated by TGF-β family members [19]. Emerging evidence suggests an oncogenic role of *PITX2* [20,21], and DNA methylation of the *PITX2* gene locus has been established as a prognostic biomarker in various human malignancies. DNA methylation of the *PITX2ab* promoter region has been associated with outcome in lung cancer [22] and hormone-receptor positive breast cancer patients [17,23–25]. Furthermore, *PITX2ab* and *PITX2c* methylation is associated with biochemical recurrence (BCR) in patients with prostate cancer after radical prostatectomy [26–29].

Recently, a long non-coding RNA (lncRNA) gene located adjacent and downstream of the *PITX2* gene region and assigned to Ensembl gene *RP11-380D23*.*2* (ENSG00000250103) was described. This gene is referred to as *PANCR* (*PITX2* adjacent noncoding RNA) and is expressed mainly in the left atrium of the human heart. Functionally, it has been suggested that *PANCR* co-regulates *PITX2c* expression during cardiomyocyte differentiation [30]. Because of numerous known functions of lncRNA in a range of cellular processes, dysregulation of lncRNAs not surprisingly has been identified as a hallmark in various benign and malignant diseases including BTC (for review see ref. [31]). Up-regulation of the lncRNAs *MALAT1* and *CCAT1*, for instance, has been shown to promote tumor progression in gallbladder cancer [32,33]. However, no direct link of *PANCR* to malignant diseases or its methylation state have been published so far.

Given the prevailing need for diagnostic, prognostic, and predictive tools in the treatment of BTC, and the emerging evidence on an involvement of *PITX2* in various cancers, this study was designed to investigate the DNA methylation status of the promoter regions of the *PITX2* gene loci and the intragenic region of lncRNA *PANCR* in BTC. Furthermore, we sought to assess a possible clinical association of such epigenetic alterations for potential future biomarker implications.

## Material and Methods

### Ethical Approval

The present study has been approved by the Institutional Review Board of the University Hospital Bonn, which waived the need for written informed consent from the participants.

### Patients and Sample Collection

The patient cohort included 80 individuals who had undergone surgical resection for gallbladder carcinoma, intrahepatic cholangiocarcinoma (CC), perihilar CC, and extrahepatic CC at the Department of General, Visceral, Thoracic, and Vascular Surgery, University Hospital Bonn between 12/1989 and 05/2014 (clinicopathological parameters are summarized in Table 1). Archival formalin-fixed and paraffin-embedded tissue (FFPET) blocks were obtained from each patient and representative hemotoxylin and eosin (HE) slides taken from each tumor. Subsequently, an area of adenocarcinoma and if available a region with entirely normal adjacent tissue (NAT) was identified by experienced gastrointestinal pathologists at the Institute of Pathology, University of Bonn. A semi-automatic Tissue Microarrayer instrument with 0.6 mm stainless steel needle punchers was used to obtain tissue from FFPET blocks. Two punch biopsies of 0.6 mm diameter were taken from the most representative area of both tumor and NAT tissue.

**Table 1:** Clinicopathological parameters of 80 biliary tract cancer patients.

		m*PITX2ab*^†^		m*PITX2c*^†^		m*PANCR*	
Variable	No. of Patients (%)	Mean [%] ± SD	*p*-value	Mean [%] ± SD	*p*-value	Mean [%] ± SD	*p*-value
**Follow-Up**							
Mean / Median [months]	21.3 / 15.0						
Range [months]	0–104						
Deceased [n]	50 (62.5%)	29.8 ± 34.5	0.47 ¥	32.5 ± 29.2	0.33 ¥	9.0 ± 16.5	0.08 ¥
Censored [n]	30 (37.5%)	33.5 ± 35.8		29.1 ± 33.6		14.9 ± 20.5	
**Age at OP**			0.97 #		0.45 #		0.97 #
≤ 60 years	37 (46.3%)	35.2 ± 38.1		32.9 ± 30.5		10.1 ± 19.0	
> 60 years	43 (53.8%)	27.7 ± 31.8		29.7 ± 31.4		12.1 ± 17.6	
Mean/ Median [years]	61.8 / 63.0						
Range [years]	38–83						
**Gender**			0.37 ¥		0.08 ¥		0.68 ¥
Female	31 (38.8%)	35.5 ± 34.1		36.9 ± 27.8		12.8 ± 18.8	
Male	49 (61.3%)	28.3 ± 35.3		27.4 ± 32.4		10.2 ± 17.9	
**Tumor location**			0.25 §		0.010 § *		0.16 §
Gallbladder	17 (21.3%)	35.3 ± 36.5		36.3 ± 30.1		16.6 ± 21.4	
Intrahepatic CC	30 (37.5%)	38.5 ± 38.8		37.9 ± 30.3		15.4 ± 22.4	
Extrahepatic CC	7 (8.8%)	9.3 ± 11.8		6.3 ± 30.0		2.9 ± 3.6	
Perihiliar (Klatskin tumor)	22 (27.5%)	26.6 ± 29.0		25.9 ± 30.0		4.5 ±6.8	
Unknown	4 (5.0%)						
**T stage**			0.93 #		0.51 #		0.41 #
T1	12 (15.0%)	33.3 ± 37.4		36.9 ± 34.9		21.7 ± 29.9	
T2	19 (23.8%)	30.6 ± 31.2		34.6 ± 29.8		7.5 ± 12.8	
T3	39 (48.8%)	32.5 ± 36.9		26.6 ± 28.5		10.2 ± 16.4	
T4	4 (5.0%)	18.1 ± 17.1		45.7 ± 35.0		12.3 ± 10.7	
Unknown	6 (7.5%)						
**Lymphe node status**			0.44 ¥		0.09 ¥		0.38 ¥
N0	34 (42.5%)	25.1 ± 30.6		23.4 ± 28.9		8.1 ± 14.6	
N1, N2	18 (22.5%)	30.4 ± 29.9		37.0 ± 31.6		8.0 ± 10.7	
Unknown	28 (35.0%)						
**Distant metastases**			0.39 ¥		0.19 ¥		0.14 ¥
M0	63 (78.8%)	30.1 ± 34.6		27.3 ± 29.2		11.5 ± 19.4	
M1	7 (8.8%)	26.3 ± 35.0		44.5 ± 40.5		4.6 ± 9.0	
Unknown	10 (12.5%)						
**Tumor grade**			0.78 #		0.75 #		0.58 #
G1	5 (6.3%)	24.3 ± 25.6		24.1 ± 14.6		19.3 ± 34.7	
G2	38 (47.5%)	30.7 ± 35.9		31.7 ± 31.1		13.1 ± 20.1	
G3	33 (41.3%)	30.9 ± 33.6		29.8 ± 31.2		8.0 ± 12.1	
Unknown	4 (5.0%)						
**Surgical margin**			0.28 ¥		0.13 ¥		0.66 ¥
R0	56 (70.0%)	28.4 ± 33.9		28.2 ± 31.6		10.6 ± 17.5	
R1	20 (25.0%)	25.6 ± 34.4		36.2 ± 25.4		13.3 ± 20.9	
Unknown	4 (5.0%)						

*P* values refer to

¥ = Mann-Whitney-U

# = Spearman’s *ρ*

§ = Kruskal-Wallis

* = statistically significant

^†^ m*PITX2ab*: methylation of the promoter region of transcript variants A and B; m*PITX2c*: methylation of the alternative promoter region of transcript variant C

SD: standard deviation

### Sample and Calibrator DNA Preparation

FFPET cores were deparaffinized, lysed, and bisulfite-converted using the innuCONVERT Bisulfite All-In-One kit (Analytik Jena) according to the manufacturer’s recommendations [34]. The DNA concentration was determined by UV spectrophotometry using 33 as multiplication factor for single stranded bisulfite DNA (Nanodrop® ND-1000 spectral photometer [Thermo Scientific]). A calibrator sample of bisulfite-converted artificially methylated human DNA was prepared as described previously [22].

### Quantitative Real-time PCR Assays

DNA methylation of *PITX2* and *PANCR* was quantified using two different real-time PCR-based methodologies. *PITX2* methylation was assessed with a quantitative methylation-specific PCR (qMSP). qMSP primers cover CpG-sites and specifically amplify methylated alleles. The quantification of total DNA was achieved with primers targeting a locus that does not contain CpG-sites and therefore amplifying alleles irrespective of the methylation status. In contrast, *PANCR* methylation was determined using a quantitative methylation (QM) assay with primers that do not contain CpG-sites within the target region and therefore amplify methylated as well as unmethylated sequences. The CpG-sites located in between the primer binding sites are probed with two hydrolysis probes which specifically and competitively detect methylated or unmethylated alleles, respectively.

The relative DNA methylation of *PITX2ab*, *PITX2c*, and *PANCR* was quantified in triplicate qPCR reactions using an AB 7500 Fast Real-Time PCR System (Life Technologies Corporation). The PITX2ab-assay was conducted as previously described [35]. The PITX2c- and PANCR-assay were performed in 20-μl reactions containing 0.25 mM of each dNTP, 1 x PCR buffer [35 mM Tris (pH 8.4), 50 mM KCl, 6 mM MgCl_2_, 4% Glycerol], 3 U FastStart Taq DNA polymerase (Roche), 0.006 μl ROX solution (prepared as previously described in ref. [22]), and 50 ng (according to UV quantification) bisulfite-converted sample DNA or maximal 5 μl. The PITX2c-assay contained 0.4 μM of each primer (forward: TTTGCGGCGGTAGTAGTC, reverse: AAAAATATACGTATACGCGTTA), 0.3 μM detection probe (ATTO647N-CGACGCGGTTTTTTGAGC-BHQ-2), 0.23 μM of each PITX2-reference primer (forward: TTGGTGATTAATTTAAAGGAGTTAT, reverse: AATTACCTAAAAACCAAACCTAA), and 0.3 μM PITX2-reference detection probe (R6G-TTAAAGAAATGGTGAGAGTTTGGTAT-BHQ-1). The PANCR-assay contained 0.4 μM of each primer (forward: TCCAATCCCTCATTTATCC, reverse: AATTTTTTGGAGGTTATTTATT) and 0.3 μM of each detection probe (methylated: 6-FAM-CGCTTCCTACGACTAAACGA-BHQ-1, unmethylated: HEX-CACTTCCTACAACTAAACAATCCT-BHQ-1). The amplification was carried out using following temperature profile: 15 min at 95°C of initial denaturation followed by 50 cycles with 75 sec at 60°C (for PITX2c-assay) or 60 sec at 56°C (for PANCR-assay; for both 100% ramp rate), and 15 sec at 95°C (75% ramp rate).

For all qPCR assays, the following threshold and baseline settings were used: 0.007 (threshold PITX2ab), 0.01 (threshold PITX2c, PITX2-reference, PANCR), and 3–20 (baseline). For each sample, the relative methylation was determined using the ΔΔCT method [36] as follows: ΔΔCT_Sample_ = ΔCT_Sample_ – ΔCT_Calibrator_, where ΔCT_Sample_ = CT_PITX2 / methylated_ – CT_reference / unmethylated_ and ΔCT_Calibrator_ = CT_PITX2 / methylated_ – CT_reference / unmethylated_. For the PITX2ab- and PITX2c-assay, 5 ng of bisulfite-converted artificially methylated human DNA was used as calibrator sample [22] and percentage methylation was calculated using the formula: Methylation = 100 × 2^-ΔΔCT^. The relative methylation of *PANCR* was determined by the use of a 5 ng calibrator sample consisting of 50% artificially methylated and 50% unmethylated bisulfite-converted DNA. Percentage methylation was calculated using the formula: Methylation = 100 / (1 + 2^ΔΔCT^). Invalid qPCR results indicated by high CT values (CT_reference_ or CT _methylated / unmethylated_ > 36) were omitted from analysis. Mixtures of methylated and unmethylated bisulfite-converted DNA were used for the evaluation of the PITX2c- and PANCR-assay performance. Each methylation mixture (0, 0.8, 1.6, 3.1, 6.3, 12.5, 25, 50, 75, and 100%) contained artificially methylated bisulfite-converted DNA in unmethylated bisulfite-converted DNA from sperm (5 ng total DNA template) and was measured in triplicates.

### Statistical Analysis

DNA methylation levels of malignant tissue and NAT samples were compared using Wilcoxon signed rank tests for paired samples. Spearman’s *ρ* was performed for bivariate correlation analysis. Mann-Whitney-U and Kruskal-Wallis were used to evaluate differences between DNA methylation and clinicopathological parameters. Univariate Cox proportional hazard analysis, Kaplan-Meier analysis, and log-rank test were conducted to investigate overall survival (OS). Multivariate Cox proportional hazard analysis with backward elimination (Wald test) was performed to assess the prognostic value of DNA methylation. Only variables that were statistically significant by univariate analysis were included. Overall survival time indicated the time between the date of primary tumor resection and the date of death or the last patient contact, respectively. Statistical analysis was performed using SPSS Statistics 22 (IBM) and GraphPad Prism 4 (GraphPad Software). All reported *p* values were two-tailed and *p* values < 0.05 were considered statistically significant.

## Results

### Analyzed Genomic Locations within *PITX2* Gene Region and Analytical Assay Performance

DNA methylation within the *PITX2* gene and its adjacent ncRNA *PANCR* were determined at three distinct genomic locations: promoters of the transcript variants *PITX2*a/b and *PITX2c* as well as in an intragenic GC-rich region located in an annotated promoter flanking site within the *PANCR* gene body (Fig 1).

**Fig 2:**
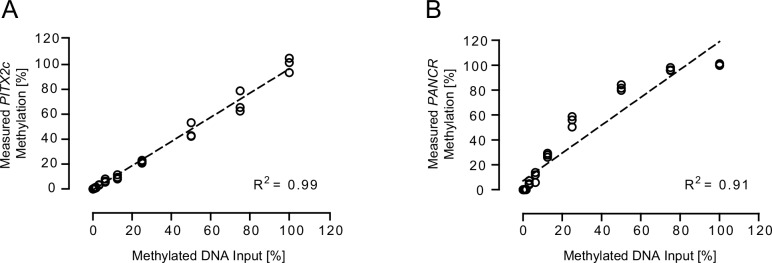
Analytical performances of PITX2c- and PANCR-assays. Standard curve for (A) PITX2c- and (B) PANCR-assay shown as a plot of measured methylation values (%) for a mixture of bisulfite-converted methylated DNA in bisulfite-converted unmethylated DNA. Each mixture was analyzed in triplicates and each data point reflects the methylation result from one single PCR.

The analytical performance of the *PITX2c* and *PANCR* DNA methylation assays were verified using mixtures of methylated and unmethylated bisulfite-converted DNA from sperm, which is known to be unmethylated at many loci [37]. A plot of measured methylation values vs. amount of methylated DNA input (100% - 0%) demonstrated high linearity for both PITX2c- (R^2^ = 0.99) and PANCR-assay (R^2^ = 0.91). The assays were sensitive down to 0.8% methylated DNA (equivalent to 40 pg) in a total of 5 ng bisulfite-converted DNA (Fig 2). The analytical performance of the PITX2ab-assay has been described in detail earlier [35].

### *PITX2ab* and *PITX2c* Promoter DNA Methylation in Tumor and Normal Tissue of Biliary Tract Cancers

**Fig 3:**
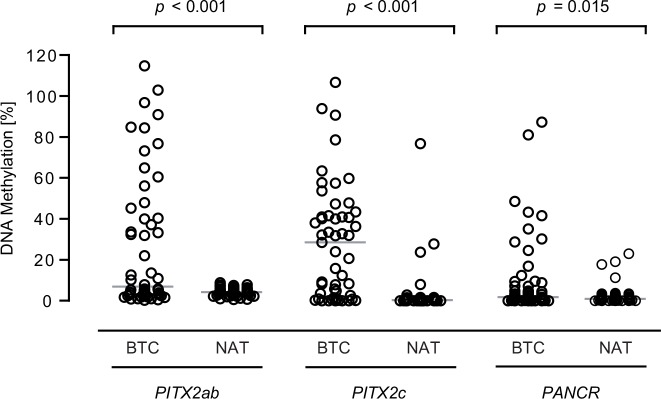
*PITX2* and *PANCR* DNA methylation is significantly increased in BTC tumor samples compared to matched NAT. The light grey horizontal indicates the median value. Measurement contained matched samples for *PITX2ab* (n = 52), *PITX2c* (n = 51), and *PANCR* (n = 56). *P* values refer to Wilcoxon signed rank test.

Relative DNA methylation in the *PITX2ab* promoter region was significantly increased in tumor tissue (median 6.9%) compared to 52 matched NATs (median 4.2%, *p* < 0.001; Fig 3). In the *PITX2c* promoter region, the methylation difference between 51 matched tumor (median 28.5%) and NAT (median 0.4%) samples was even more pronounced (*p* < 0.001). The measured *PANCR* methylation significantly differed between 56 tumor (median 1.7%) and NAT (median 0.9%) specimens (*p* = 0.015).

### Correlation of *PITX2* and *PANCR* DNA Methylation

Promoter methylation of the *PITX2ab* variant positively correlated with *PITX2c* (Spearman’s *ρ* = 0.333; *p =* 0.003; n = 78) in tumor samples as well as in NAT specimens (Spearman’s *ρ* = 0.345, *p =* 0.014, n = 50). In tumor specimens, intragenic *PANCR* methylation significantly correlated with *PITX2ab* (Spearman’s *ρ* = 0.390; *p* < 0.001; n = 80) but not *PITX2c* (Spearman’s *ρ* = 0.139; *p* = 0.23; n = 78), whereas *PANCR* methylation was significantly associated with *PITX2c* methylation in NAT (Spearman’s *ρ* = 0.328; *p* = 0.016; n = 53) but did not reach statistical significance for *PITX2ab* (Spearman’s *ρ* = 0.269; *p* = 0.054; n = 53).

### Association of *PITX2* and *PANCR* DNA Methylation with Clinicopathological Parameters

With respect to clinicopathologic parameters, only *PITX2c* methylation differed significantly depending on tumor location (extrahepatic CC vs. gallbladder [*p* = 0.029] and vs. intrahepatic CC [*p* = 0.015], respectively) (Table 1). Additional statistically significant associations were not found.

### Association of *PITX2* and *PANCR* DNA Methylation with Overall Survival

As *PITX2* and *PANCR* methylation were increased in tumor tissue, their potential as prognostic factor for OS was investigated by means of univariate Cox regression and Kaplan-Meier analysis.

**Fig 4:**
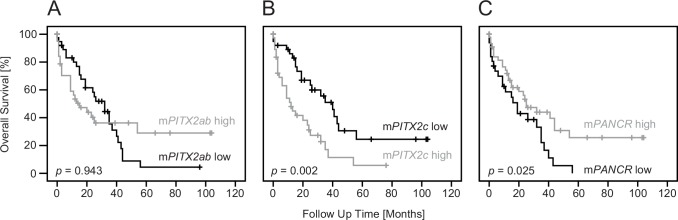
Kaplan–Meier plots comparing overall survival (OS) of biliary tract cancers patients after resection. (A) Dichotomized *PITX2ab* DNA methylation, stratified into low (black line, n = 40) and high (grey line, n = 40) levels had no significant impact on OS. (B) However, patients with low *PITX2c* DNA methylation (black line, n = 39) had a significantly better OS compared to patients with high methylation (grey line, n = 39). (C) Conversely, low *PANCR* methylation (black line, n = 33) associated with worse prognosis and high methylation (grey line, n = 47) significantly improved OS.

After dichotomization using a median cut-off (10.8%), *PITX2ab* methylation was not associated with OS in univariate Cox regression (hazard ratio [HR] 1.02, *p* = 0.94, 95% confidence interval [CI]: 0.58–1.79) or Kaplan-Meier analysis (Fig 4A). In contrast, patients with high levels of *PITX2c* DNA methylation had an increased risk of death in univariate Cox regression analysis (HR 2.48, *p* = 0.002, 95% CI: 1.38–4.48). Patients with *PITX2c* DNA methylation levels below the median cut-off (31.8%) had an increased median survival time of up to 40 months (median value, 95% CI: 28.4–51.6) compared to 12 months for patients with higher *PITX2c* DNA methylation (median value, 95% CI: 5.5–18.5) (Fig 4B). In univariate Cox regression analysis, the risk of death was almost half as high for patients with higher *PANCR* methylation (dichotomized cut-off 1.6%; HR 0.54, *p* = 0.030, 95% CI: 0.30–0.94). Consequently, low *PANCR* methylation was associated with worse prognosis and reduced median survival time (19 months) compared to increased methylation (25 months) in Kaplan-Meier analysis (Fig 4C).

In addition, differences in OS outcome were revealed for the clinicopathological parameters nodal status (pN) and surgical margin (R) in univariate Cox regression analysis (Table 2). In multivariate Cox regression analysis, however, *PITX2c* DNA methylation lost its prognostic power as opposed to *PANCR* methylation and the known predictive parameters pN and R.

**Table 2:** Results from univariate and multivariate Cox proportional hazard analyses of overall survival.

	Univariate Cox		Multivariate Cox	
Clinicopathological parameter / biomarker	Hazard ratio [95% CI]	*p*-value	Hazard ratio [95% CI]	*p*-value
Tumor grade (G3 vs. G1/G2)	1.28 [0.73–2.24]	0.38		
Tumor stage (T3/T4 vs. T1/T2)	1.06 [0.60–1.86]	0.85		
Nodal status (N1/N2 vs. N0)	2.25 [1.08–4.72]	0.031	2.32 [1.08–4.98]	0.031
Distant metastases (M1 vs. M0)	2.32 [0.96–5.59]	0.062		
Surgical margin (R1 vs. R0)	3.09 [1.63–5.84]	0.001	2.98 [1.31–6.74]	0.009
*PITX2ab* methylation (high vs. low)	1.02 [0.58–1.79]	0.94		
*PITX2c* methylation (high vs. low)	2.48 [1.38–4.48]	0.002	1.28 [0.51–3.19]	0.60
*PANCR* methylation (high vs. low)	0.54 [0.30–0.94]	0.030	0.42 [0.20–0.88]	0.021

CI: Confidence interval.

## Discussion

BTC is a rare but clinically challenging disease with a poor prognosis. Due to non-specific symptoms in approx. 90% of patients presenting with BTC, the disease is often diagnosed in a locally advanced or metastatic and unresectable state. Complete surgical resection is the only curative treatment and applicable in only 10% of patients with early stage disease [38]. Even patients with localized BTC, who have undergone resection with intent to cure, have a high risk for disease recurrence [39]. A combination chemotherapy of gemcitabine and cisplatin is the standard-of-care for advanced and recurrent BTCs. This regimen, however, only has a small survival benefit compared to untreated patients with overall survival rates and progression-free survival of approx. 12 months and 8 months, respectively [40].

The present study demonstrated aberrant DNA hypermethylation of *PITX2* promoter regions in tumor specimens of BTC compared to normal tissue. Furthermore, patients with *PITX2c* promoter hypermethylation revealed a significantly reduced OS, albeit OS did not qualify as an independent prognostic factor. This observation, even if only detected in univariate analysis in our small cohort, might be supported by a report which demonstrated that *PITX2* is functionally involved in the suppression of pancreatic cancer progression. In pancreatic ductal adenocarcinoma (PDAC), the *TGF-β* signaling factor *SMAD4* was shown to stimulate *PITX2* expression, and a strong immunohistochemical PITX2 staining was reported to be associated with a better prognosis, whereas no correlation with methylation was observed [41]. However, in this study methylation was not measured in *PITX2* promoter regions. Nevertheless, it is generally assumed that hypermethylation of a promoter region leads to transcriptional gene silencing [42,43]. *Vice versa*, low methylation would lead to a normal level of gene expression. The finding that patients with improved survival revealed *PITX2c* promoter hypomethylation might be caused by elevated *PITX2c* expression compared to patients of the hypermethylation group. Furthermore, *PITX2c* promoter is known to be regulated by TGF-β signaling [19], and SMAD4 protein binding was also reported for PDAC [41]. As biliary tract and pancreatic lesions have similar molecular and histological features [44], it might be admissible to transfer these conclusion to BTC. This theory will ultimately have to be proven by future investigations of expression levels though.

In contrast to our findings on *PITX2c*, *PANCR* hypermethylation revealed a significantly improved OS and retained its predictive power in univariate as well as multivariate Cox proportional hazard analysis. However, the *PANCR* DNA methylation was examined adjacent to a CpG island within an intragenic region instead of a promoter region. A positive correlation of hypermethylated gene body with an increased gene expression has been reported by several groups [45–47]. One might infer that *PANCR* expression is elevated as well as *PITX2c*; besides, a positive correlation of expression was observed in human adult left atria tissue [30].

In spite of the presented possible biomarker capabilities, it becomes apparent that aberrant DNA methylation of *PITX2* variants is not as prognostic for BTCs as it is for breast or prostate cancer [17,35]. In the latter, these markers may identify patients with an adverse clinical courses who might actually benefit from a radical treatment, while patients with a better prognosis might benefit from a more conservative treatment with fewer side effects. Due to delayed diagnoses and a generally poor prognosis of BTC, all patients included actually underwent a radical therapy (i.e. complete surgical resection, chemotherapy). For clinical decision-making, predictive biomarkers are desired to identify patient subpopulations that most likely benefit from chemotherapy or molecular targeting therapy. Last but not least, a basic science rationale for studying *PITX2* and *PANCR* methylation was to gain new insight into the biology of BTC in order to find novel therapeutic implications in this lethal disease.

The study has some potential limitations: The low incidence of BTC allows the inclusion of only a small cohort that was analyzed in a retrospective fashion, clearly limiting the power of statistical analysis as well as lowering the evidence level. Even if the biliary ductal system shares one embryonic background, previous studies have documented distinct histomorphological and molecular patterns referring to the location of the lesion within the biliary tree [5,6]. In addition, the present study does not unveil the biological significance of *PITX2* and *PANCR* methylation during biliary tract carcinogenesis and disease progression. An association between DNA methylation of the analyzed loci with mRNA and protein expression of the respective gene products need to be conducted in order to elucidate any direct epigenetic control of the genes *via* DNA methylation. Furthermore, cell culture experiments with hypomethylating agents (i.e. 5-azacytidine) should be performed in order to investigate the functional role of *PITX2* and *PANCR*. Eventually, the potential value of *PITX2* and *PANCR* methylation as predictive biomarkers for particular therapies (i.e. targeted therapies, epigenetic therapies, immunotherapies, chemotherapies) needs to be tested in preclinical models.

## Conclusions

Assessment of *PITX2* and *PANCR* methylation in BTC revealed a possible new biomarker implication to help differentiate tumors from normal tissue as well as to identify distinct biologic behaviours of individual tumors for a possible future therapeutic stratification. The data have to be evaluated on a prospective level. Herein, an appropriate stratification into BTC subtypes seems inevitable.
